# Implicit symmetric and symplectic exponentially fitted modified Runge–Kutta–Nyström methods for solving oscillatory problems

**DOI:** 10.1186/s13660-018-1915-4

**Published:** 2018-11-20

**Authors:** Bing Zhen Chen, Wen Juan Zhai

**Affiliations:** 1 School of Science, Beijing Jiaotong University, Beijing, China; 2 Department of Mathematics, Beijing Jiaotong University Haibin College, Cangzhou, China

**Keywords:** Implicit, Symmetric, Symplectic, Exponentially fitted, Modified Runge–Kutta–Nyström method, Oscillatory problem

## Abstract

Symplectic exponentially fitted RK and RKN methods have been extensively studied by many researchers. Due to their good property, they have been applied to many problems such as pendulum, Morse oscillator, harmonic oscillator, Lennard–Jones oscillator, Kepler’s orbit problem, and so on. In this paper, we construct an implicit symmetric and symplectic exponentially fitted modified Runge–Kutta–Nyström (ISSEFMRKN) method. The new integrator integrates exactly differential systems whose solutions can be expressed as linear combinations of functions from the set $\{\exp(\lambda t),\exp(-\lambda t)\}$, $\lambda\in\mathbb{C}$, or equivalently $\{\sin(\omega t),\cos(\omega t)\}$ when $\lambda=i\omega$, $\omega \in\mathbb{R}$. When $z=\lambda h$ approaches zero, the ISSEFMRKN method reduces to the classical symplectic, symmetric RKN integrator. Numerical experiments are accompanied to show the efficiency and competence of the new method compared with some efficient codes in the literature.

## Introduction

In plenty of applied sciences such as celestial mechanics, astrophysics, chemistry, electronics, molecular dynamics, and so forth, the following second-order ODEs initial value problems (IVP) often arise:

1 $$ y''=f(t,y), \quad\quad y(t_{0})=y_{0},\quad\quad y'(t_{0})=y'_{0}, \quad t\in[0,t_{\mathrm{end}}], $$

whose solutions exhibit an oscillatory character. Such problems are of great interest and have been studied extensively. Roughly speaking, there are two categories of approaches to numerical integration of the IVP (1): indirect and direct. In the first place, if a new variable *v* is introduced to represent the first derivative $y'$, then (1) can be transformed into a partitioned system of first-order equations

$$ y'=v,\quad\quad v'=f(t,y),\quad\quad y(x_{0})=y_{0},\quad\quad v(t_{0})=y'_{0}. $$

This new reformulated problem can be solved by the general Runge–Kutta (RK) methods, partitioned Runge–Kutta (PRK) methods, or two-step methods (see Refs. [5, 6, 10, 19, 20, 25, 26, 29, 32, 33]). In the second place, the Runge–Kutta–Nyström (RKN) method, which was introduced by Nyström in 1925, is designed to handle the second-order problem (1) directly. The detailed information can be seen in [4]. From then on, there have been many researchers focused on the RKN method. Subsequently, a lot of variations of RKN methods were given, such as [27, 28, 31, 34] and others. The research on RK, RKN is tremendous, but it mainly focuses on explicit methods due to easier coding and less time consuming in comparison to implicit methods. The implicit methods are more suitable for solving stiff ODEs than the explicit methods. There are some researchers who work on the implicit RKN methods, such as [16–18, 22, 24].

If the solutions of ODEs satisfy a conservation law, such as dynamical systems for which total energy is conserved, the symplectic methods [8, 9, 30] should be considered. The term symplectic essentially means area preserving in a phase space. Approximate solutions generated by symplectic methods are conservative even at finite resolution, in contrast with numerical methods that generate approximate solutions that are conservative only in the limit as the time step size approaches zero. Symplectic methods have been applied to many problems such as pendulum, Morse oscillator, harmonic oscillator, Lennard–Jones oscillator, Kepler’s orbit problem, and so on. As pointed out in Chap. V and Chap. XI of [13], symmetric methods show a better long time behavior than non-symmetric ones when solving the reversible differential system. So, some symmetric and symplectic RKN methods are proposed such as [24, 34].

During the last thirty years, many researchers have been working on exponentially fitted RK or RKN methods. This technique was first analyzed in theory by Gautschi [12] and Lyche [21]. Exponentially fitted methods which intend to integrate exactly differential systems whose solutions can be expressed as linear combinations of functions from $\{\exp(\lambda t),\exp(-\lambda t), \lambda\in\mathbb{C}\}$, or equivalently, from $\{\sin(\omega t ),\cos(\omega t), \omega\in\mathbb{R}\}$ with $\lambda=i\omega$, $i^{2}=-1$, share better behaviors when applied to oscillatory problems than non-symplectic methods. Therefore, it has become an indispensable tool for solving oscillatory problems. The construction of exponentially fitted RK(N) methods is originally due to Paternoster [23], and a detailed exposition of exponentially fitted methods with an extensive bibliography on this subject can be found in Ixaru and Vanden Berghe [15].

Recently, the authors [35] have given a two-stage implicit symmetric and symplectic exponentially fitted Runge–Kutta–Nyström method (ISSEFRKN). It shows a good behavior compared with some existing methods. Exactly, this method is not a complete exponential fitting method. It can be seen from the process of derivation that there are two different expressions of $b_{1}$. So the authors make them as close as possible by choosing a parameter $\theta=\pm\frac{\sqrt{3}}{6}$. To avoid this happening, we investigate the construction of two-stage implicit symmetric and symplectic exponentially fitted modified Runge–Kutta–Nyström (ISSEFMRKN). Compared with the ISSEFRKN method, we add modified parameters for the term *h* in the internal stages. Consequently, we can obtain unique expression of every coefficient which is not true for ISSEFRKN. The new method ISSEFMRKN also reduces to the classical symplectic, symmetric RKN integrator when the parameter *z* approaches zero.

This paper is organized as follows: In Sect. 2 we present the notations and definitions to be used in the rest of the paper as well as some previous results on symmetric and symplectic methods. In Sect. 3 we make a study of the local truncation error, obtaining the order conditions (up to fifth order) for this class of methods. In Sect. 4, we derive the new two-stage implicit symplectic and symmetric EFMRKN integrator based on the former conditions. In Sect. 5, we devote to some numerical experiments. The numerical results show that the new method is more accurate and efficient compared with some other implicit RKN integrators. Finally, Sect. 6 involves in some conclusions.

## Conditions for symmetry, symplecticity, exponential fitting of modified RKN methods

In this paper, we deduce a class of exponentially fitted RKN methods which integrate exactly second-order differential systems whose solutions can be expressed as linear combinations of the set of functions $\{\exp (\lambda t),\exp(-\lambda t), \lambda\in\mathbb{C}\}$, or equivalently, from $\{\sin(\omega t ),\cos(\omega t), \omega\in\mathbb{R}\}$ with $\lambda=i\omega$, $i^{2}=-1$. This means that the internal stage and the final stage have to integrate exactly these sets of functions. In order to do so, we must introduce some modifications to the ordinary RKN scheme. Here we consider the following *s*-stage modified implicit RKN method for the second-order ODEs (1):

2 $$ \textstyle\begin{cases} Y_{i}=y_{0}+c_{i}\gamma_{i}h{y'_{0}}+h^{2}\sum_{j=1}^{s}a_{ij}f(t_{0}+c_{j}h,Y_{j}),\quad i=1,\ldots,s, \\ y_{1}=y_{0}+h{y'_{0}}+h^{2}\sum_{i=1}^{s}\bar {b}_{i}f(t_{0}+c_{i}h,Y_{i}), \\ y'_{1}={y'_{0}}+h\sum_{i=1}^{s}b_{i}f(t_{0}+c_{i}h,Y_{i}), \end{cases} $$

which can be expressed in the Butcher tableau as follows:

**Table:** 

*c*	e *γ*	A
	1 1	$\bar{b}^{T}$
	1	$b^{T}$

=

**Table:** 

$c_{1}$	1	$\gamma_{1}$	$a_{11}$	⋯	$a_{1s}$
⋮	⋮	⋮	⋮	⋱	⋮
$c_{s}$	1	$\gamma_{s}$	$a_{s1}$	⋯	$a_{ss}$
	1	1	$\bar{b}_{1}$	⋯	$\bar {b}_{s}$
		1	$b_{1}$	⋯	$b_{s}$

. It should be noted that scheme (2) coincides with the classical *s*-stage RKN formula when the coefficients $\gamma_{i}=1$, $i=1,\ldots,s$, and the remaining coefficients are constant.

Now, we set out to derive the three corner stones to construct our method. In the following subsections, we denote a one-step method for second-order ODEs (1) as $\varPhi_{h}: (y_{0},y'_{0})^{\mathrm{T}}\mapsto (y_{1},y'_{1})^{\mathrm{T}}$. Here, from $y_{0}$ to $y_{1}$, the variable goes forward with a step *h*.

### Symmetry conditions

The key to understanding symmetry is the concept of the adjoint method.

#### Definition 2.1

The adjoint method $\varPhi_{h}^{*}$ of $\varPhi_{h}$ is the inverse map of the original method with reversed time step −*h*, i.e., $\varPhi_{h}^{*}:=\varPhi_{-h}^{-1}$. In other words, $y_{1}=\varPhi_{h}^{*}(y_{0})$ is implicitly defined by $\varPhi_{-h}(y_{1})=y_{0}$. A method for which $\varPhi_{h}^{*}=\varPhi_{h}$ is called symmetric.

In this paper we consider scheme (2) whose coefficients are functions of *z*, as we do for exponentially fitted type methods [32, 33]. Then the conditions for methods (2) to be symmetric are given by the following lemma.

#### Lemma 1

*The modified RKN method* (2) *is symmetric if its coefficients satisfy the following conditions*:

3 $$\begin{aligned} &c_{i}(z)=1-c_{s+1-i}(z), \\ &c_{i}(z)\gamma_{i}(z)=1-c_{s+1-i}(z) \gamma_{s+1-i}(z), \\ &\bar{b}_{i}(z)=b_{s+1-i}(z)-\bar{b}_{s+1-i}(z), \\ &b_{i}(z)=b_{s+1-i}(z), \\ &a_{ij}(z)=a_{s+1-i,s+1-j}(z)- c_{s+1-i}\gamma _{s+1-i}(z)b_{s+1-j}(z) + b_{s+1-j}(z)-\bar{b}_{s+1-j}(z), \end{aligned}$$

*where* $z=i\omega h$, *ω* *is the principal frequency of the problem*.

#### Proof

Following the idea of Hairer et al. [14], we interchange $h\leftrightarrow-h$, $z = i\omega h \leftrightarrow-z =-i\omega h$, $t_{0} \leftrightarrow t_{0} + h$, $y_{0} \leftrightarrow y_{1}$, and $y'_{0} \leftrightarrow y'_{1}$ in formula (2), then we obtain the following equations:

4 $$ \textstyle\begin{cases} Y_{i} = y_{1}-c_{i}\gamma_{i}(z)h{y'_{1}}+h^{2}\sum_{j=1}^{s}a_{ij}(z)f(t_{0}+(1-c_{j})h,Y_{j}), \quad i=1,\ldots,s, \\ y_{0} = y_{1}-h{y'_{1}}+h^{2}\sum_{i=1}^{s}\bar {b}_{i}(z)f(t_{0}+(1-c_{i})h,Y_{i}), \\ y'_{0} = {y'_{1}}-h\sum_{i=1}^{s}b_{i}(z)f(t_{0}+(1-c_{i})h,Y_{i}). \end{cases} $$

Note that, we do not change *z* to −*z*, because we will derive exponentially fitted methods for which its coefficients are even functions of *z*. From equation (4), one can easily obtain

5 $$\begin{aligned}& y'_{1} = y'_{0} + h\sum _{i=1}^{s}b_{i}(z)f\bigl(t_{0}+(1-c_{i})h,Y_{i} \bigr) \\& \hphantom{y'_{1}}= y'_{0} + h\sum _{i=1}^{s}b_{s+1-i}(z)f\bigl(t_{0}+(1-c_{s+1-i})h,Y_{s+1-i} \bigr), \end{aligned}$$

6 $$\begin{aligned}& y_{1} = y_{0} + hy'_{1} - h^{2}\sum_{i=1}^{s}\bar {b}_{i}(z)f\bigl(t_{0}+(1-c_{i})h,Y_{i} \bigr) \\& \hphantom{y_{1}} = y_{0} + hy'_{1} - h^{2}\sum_{i=1}^{s}\bar {b}_{s+1-i}(z)f\bigl(t_{0}+(1-c_{s+1-i})h,Y_{s+1-i} \bigr) \\& \hphantom{y_{1}} = y_{0} + h \Biggl( y'_{0} + h \sum_{i=1}^{s}b_{s+1-i}(z)f \bigl(t_{0}+(1-c_{s+1-i})h,Y_{s+1-i}\bigr) \Biggr) \\& \hphantom{y_{1}}\quad{} - h^{2}\sum_{i=1}^{s} \bar {b}_{s+1-i}(z)f\bigl(t_{0}+(1-c_{s+1-i})h,Y_{s+1-i} \bigr) \\& \hphantom{y_{1}}= y_{0} + hy'_{0} +h^{2}\sum_{i=1}^{s} \bigl( b_{s+1-i}(z)-\bar {b}_{s+1-i}(z) \bigr) f\bigl(t_{0}+(1-c_{s+1-i})h,Y_{s+1-i} \bigr). \end{aligned}$$

Inserting equations (5) and (6) into $Y_{s+1-i}$, we have

7 $$\begin{aligned} Y_{s+1-i} &= y_{1}-c_{s+1-i}\gamma_{s+1-i}(z)h{y'_{1}} \\ & \quad{} + h^{2}\sum_{j=1}^{s}a_{s+1-i,s+1-j}(z)f \bigl(t_{0}+(1-c_{s+1-j})h,Y_{s+1-j}\bigr) \\ &= y_{0} + hy'_{0}+ h^{2}\sum _{j=1}^{s} \bigl( b_{s+1-j}(z)- \bar{b}_{s+1-j}(z) \bigr) f\bigl(t_{0}+(1-c_{s+1-j})h,Y_{s+1-j} \bigr) \\ & \quad{} -c_{s+1-i}\gamma_{s+1-i}(z)h \Biggl( y'_{0} + h\sum_{j=1}^{s}b_{s+1-j}(z)f \bigl(t_{0}+(1-c_{s+1-j})h,Y_{s+1-j}\bigr) \Biggr) \\ & \quad{} + h^{2}\sum_{j=1}^{s}a_{s+1-i,s+1-j}(z)f \bigl(t_{0}+(1-c_{s+1-j})h,Y_{s+1-j}\bigr) \\ &= y_{0} + \bigl( 1-c_{s+1-i}\gamma_{s+1-i}(z) \bigr) hy'_{0} \\ & \quad{} + h^{2}\sum_{j=1}^{s} \bigl( a_{s+1-i,s+1-j}(z)- c_{s+1-i}\gamma_{s+1-i}(z)b_{s+1-j}(z) \\ & \quad{} + b_{s+1-j}(z)-\bar{b}_{s+1-j}(z) \bigr) f \bigl(t_{0}+(1-c_{s+1-j})h,Y_{s+1-j}\bigr). \end{aligned}$$

Comparing equations (7), (6), (5) with the counterpart in (2) respectively, we can obtain the symmetric conditions (3). □

Omitting the variable *z*, i.e., all the coefficients are constants, they reduce to symmetric conditions for the traditional RKN methods.

### Symplecticity conditions

Now, we turn to the symplecticity conditions for scheme (2). Symplecticity is defined for a Hamiltonian system. On many occasions, the problem under consideration takes the form of a Hamiltonian system

$$ \dot{p}=-\frac{\partial}{\partial q}U(t,q), \quad\quad \dot{q}=M^{-1}p $$

with the Hamiltonian

$$ H(t,p,q)=\frac{1}{2}p^{\mathrm{T}}M^{-1}p + U(t,q), $$

where *M* is a symmetric positive definite constant matrix. This system is equivalent to the second-order equation (1) with $f(t,q)=-M^{-1}\frac {\partial}{\partial q}U(t,q)$. To the end of obtaining the symplectic conditions for (2), the following definition, which can be found in [14], is essential.

#### Definition 2.2

A one-step method is symplectic if, for every smooth Hamiltonian function *H* and for every step size *h*, the corresponding flow preserves the differential 2-form

$$ {\mathrm{d}}p\wedge{\mathrm{d}}q=\sum_{i=1}^{n} {\mathrm {d}}p_{i}\wedge{\mathrm{d}}q_{i}, $$

where the one-forms $\mathrm{d}p_{i}$, respectively $\mathrm {d}q_{i}$, map a tangent vector *ξ* to its *i*th, respectively $(n+i)$th, component. Here, we assume *p* and *q* all have *n* components. Furthermore, $\mathrm{d}p_{i}\wedge{\mathrm {d}}q_{i}$ is a bilinear map acting on a pair of vectors

$$\begin{aligned} {\mathrm{d}}p_{i}\wedge{\mathrm{d}}q_{i}( \xi_{1}, \xi_{2})&=\det \begin{pmatrix} {\mathrm{d}}p_{i}(\xi_{1}) & \mathrm{d}p_{i}(\xi_{2}) \\ \mathrm{d}q_{i}(\xi_{1}) & \mathrm{d}q_{i}(\xi_{2}) \end{pmatrix} \\ &=\mathrm{d}p_{i}(\xi_{1})\mathrm{d}q_{i}( \xi_{2}) - \mathrm {d}p_{i}(\xi_{2}) \mathrm{d}q_{i}(\xi_{1}) \end{aligned}$$

and satisfies Grassmann’s rules for exterior multiplication

8 $$\begin{aligned} {\mathrm{d}}p_{i}\wedge{\mathrm{d}}p_{j} = - \mathrm{d}p_{j}\wedge {\mathrm{d}}p_{i}, \quad\quad { \mathrm{d}}p_{i}\wedge{\mathrm {d}}p_{i} = 0. \end{aligned}$$

Hamiltonian systems have been seen to possess two remarkable properties:

- the solutions preserve the Hamiltonian $H(p, q)$;
- the corresponding flow is symplectic, i.e., preserves the differential 2-form $\mathrm{d}p\wedge{\mathrm{d}}q$.

As is pointed out by Feng [7], “It is natural to look forward to those discrete systems which preserve as much as possible the intrinsic properties of the continuous system.” Based on this definition, we can easily obtain the symplectic conditions for RKN formula (2).

#### Lemma 2

*The modified RKN method* (2) *is symplectic if the following conditions are satisfied*:

9 $$ \textstyle\begin{cases} \bar{b}_{i}(z)+(c_{i}\gamma_{i}(z)-1)b_{i}(z)=0, \quad i=1,\ldots,s, \\ b_{i}(z)(\bar{b}_{j}(z)-a_{ij}(z))=b_{j}(\bar{b}_{i}(z)-a_{ji}(z)), \quad i,j=1,\ldots,s. \end{cases} $$

#### Proof

Accordingly, the symplecticity of method (2) is equivalent to

10 $$ {\mathrm{d}}y_{1}\wedge{\mathrm{d}}y'_{1}= \mathrm{d}y_{0}\wedge {\mathrm{d}}y'_{0}. $$

For the left-hand side of this equation, we have

$$\begin{aligned} {\mathrm{d}}y_{1}\wedge{\mathrm{d}}y'_{1}&= \mathrm{d}y_{0}\wedge {\mathrm{d}}y'_{0}+h\sum _{i=1}^{s}b_{i}(z) \,dy_{0}\wedge{\mathrm {d}}f(Y_{i}) \\ &\quad{} +h\mathrm{d}y'_{0}\wedge {\mathrm{d}}y'_{0}+h^{2} \sum_{i=1}^{s}b_{i}(z)\mathrm {d}y'_{0}\wedge{\mathrm{d}}f(Y_{i}) \\ &\quad{} +h^{2}\sum_{i=1}^{s} \bar{b}_{i}(z)\mathrm {d}f(Y_{i})\wedge { \mathrm{d}}y'_{0}+h^{3}\sum _{i,j=1}^{s}\bar {b}_{j}(z)b_{i}(z) \mathrm{d}f(Y_{j})\wedge {\mathrm{d}}f(Y_{i}). \end{aligned}$$

Inserting $y_{0}$ in (2) to the second term of this equation, we obtain

$$\begin{aligned} {\mathrm{d}}y_{1}\wedge{\mathrm{d}}y'_{1}&= \mathrm{d}y_{0}\wedge {\mathrm{d}}y'_{0}+h^{2} \sum_{i=1}^{s}\bigl(b_{i}(z)-b_{i}(z)c_{i} \gamma_{i}(z)-\bar{b}_{i}(z)\bigr)\mathrm {d}y'_{0}\wedge{\mathrm{d}}f(Y_{i}) \\ &\quad{} +\frac{1}{2}h^{3}\sum_{i,j=1}^{s} \bigl(\bar {b}_{j}(z)b_{i}(z)-b_{i}(z)a_{ij}(z) \bigr)\mathrm{d}f(Y_{j})\wedge {\mathrm{d}}f(Y_{i}) \\ &\quad{} +\frac{1}{2}h^{3}\sum_{i,j=1}^{s} \bigl(\bar {b}_{i}(z)b_{j}(z)-b_{j}(z)a_{ji}(z) \bigr)\mathrm{d}f(Y_{i})\wedge {\mathrm{d}}f(Y_{j}). \end{aligned}$$

Therefore, considering property (8), we can obtain that (10) holds if conditions (9) are satisfied. □

### Exponential fitting conditions

Following Albrecht’s approach [2, 3], each stage of scheme (2) can be viewed as a linear multi-step method on a non-equidistant grid. With each stage one can associate a linear function as follows:

- for the internal stages,
  $$ \varphi_{i}\bigl[y(t);h;\mathbf{a}\bigr]=y(t+c_{i}h)-y(t)-c_{i} \gamma _{i}hy'(t)-h^{2}\sum _{j=1}^{s}a_{ij}y''(t+c_{j}h), \quad i=1,2,\ldots,s; $$
- for the final stages,
  $$ \textstyle\begin{cases} \varphi[y(t);h;\bar{\mathbf{b}}]=y(t+h)-y(t)-hy'(t)-h^{2}\sum_{i=1}^{s}\bar{b}_{i}y''(t+c_{i}h), \\ \varphi[y(t);h;\mathbf{b}]=y'(t+h)-y'(t)-h\sum_{i=1}^{s}b_{i}y''(t+c_{i}h). \end{cases} $$

The exponentially fitted conditions can be obtained by requiring that these functions vanish for the functions from the set $\{\exp(\pm\lambda t)| \lambda\in\mathbb{R} \text{ or } \lambda\in i\mathbb{R}\}$. The exponentially fitted conditions are stated in the following lemma.

#### Lemma 3

*The modified RKN method* (2) *is exponentially fitted if the following conditions are satisfied*:

11 $$ \textstyle\begin{cases} \sum_{j=1}^{s}a_{ij}\cosh(c_{j}z)=\frac{\cosh(c_{i}z)-1}{z^{2}}, \\ \sum_{j=1}^{s}a_{ij}\sinh(c_{j}z)=\frac{\sinh (c_{i}z)-c_{i}\gamma_{i}z}{z^{2}},\quad i=1,\ldots,s, \\ \sum_{i=1}^{s}\bar{b}_{i}\cosh(c_{i}z)=\frac{\cosh (z)-1}{z^{2}}, \sum_{i=1}^{s}\bar{b}_{i}\sinh (c_{i}z)=\frac{\sinh(z)-z}{z^{2}}, \\ \sum_{i=1}^{s}b_{i}\sinh(c_{i}z)=\frac{\cosh (z)-1}{z}, \sum_{i=1}^{s}b_{i}\cosh(c_{i}z)= \frac{\sinh(z)}{z}. \end{cases} $$

#### Proof

Requiring the internal stages vanishing when $y(t)$ taken as $e^{\lambda t}$, $e^{-\lambda t}$ respectively, we have

12 $$ \textstyle\begin{cases} e^{(\lambda t+c_{i}\lambda h)}-e^{\lambda t}-c_{i}\gamma_{i}\lambda h e^{\lambda t}-(\lambda h)^{2}\sum_{j=1}^{s}a_{ij}e^{(\lambda t+c_{j}\lambda h)}=0, \\ e^{(-\lambda t-c_{i}\lambda h)}-e^{-\lambda t}+c_{i}\gamma _{i}\lambda h e^{-\lambda t}-(\lambda h)^{2}\sum_{j=1}^{s}a_{ij}e^{(-\lambda t-c_{j}\lambda h)}=0. \end{cases} $$

Denoting $z=\lambda h$, (12) leads to

13 $$ \textstyle\begin{cases} e^{c_{i}z} -1-c_{i}\gamma_{i}z -z^{2}\sum_{j=1}^{s}a_{ij}e^{c_{j}z} =0, \\ e^{-c_{i}z}-1+c_{i}\gamma_{i}z -z^{2}\sum_{j=1}^{s}a_{ij}e^{-c_{j}z}=0. \end{cases} $$

By definitions of $\cosh(z)=(e^{z} + e^{-z})/2$ and $\sinh (z)=(e^{z}-e^{-z})/2$, equation (13) implies that

14 $$ \textstyle\begin{cases} \sum_{j=1}^{s}a_{ij}\cosh(c_{j}z)=\frac{\cosh(c_{i}z)-1}{z^{2}}, \\ \sum_{j=1}^{s}a_{ij}\sinh(c_{j}z)=\frac{\sinh (c_{i}z)-c_{i}\gamma_{i}z}{z^{2}},\quad i=1,\ldots,s. \end{cases} $$

Similarly, for the final stages, we have

$$ \textstyle\begin{cases} e^{\pm z}=1\pm z+z^{2}\sum_{i=1}^{s}\bar{b}_{i}(z)e^{\pm c_{i}z}, \\ e^{\pm z}=1\pm z\sum_{i=1}^{s}b_{i}(z)e^{\pm c_{i}z}. \end{cases} $$

It follows that

15 $$ \textstyle\begin{cases} \sum_{i=1}^{s}\bar{b}_{i}\cosh(c_{i}z)=\frac{\cosh(z)-1}{z^{2}}, \quad\quad \sum_{i=1}^{s}\bar{b}_{i}\sinh(c_{i}z)=\frac {\sinh(z)-z}{z^{2}}, \\ \sum_{i=1}^{s}b_{i}\sinh(c_{i}z)=\frac{\cosh (z)-1}{z},\quad\quad \sum_{i=1}^{s}b_{i}\cosh(c_{i}z)= \frac{\sinh(z)}{z}. \end{cases} $$

Together with (14) and (15), we obtain the desired results. □

In this paper, we say method (2) satisfies the EF conditions (14) and (15) as an exponentially fitted RKN (EFRKN) method.

## Algebraic order conditions

As we all know, a numerical method having higher algebraic order will have higher accuracy. To the end of specifying the order of the new method, we will present algebraic order conditions for exponentially fitted modified Runge–Kutta–Nystöm (EFMRKN) methods. As it occurs in the case of a classical RKN method, for an EFMRKN method, the local truncation errors in the approximations of the solution and its derivative can be expressed as

$$ \textstyle\begin{cases} e_{n+1}=y(t_{0}+h)-y_{1}=\sum_{j=1}^{p-1}h^{j+1}\sum_{i=1}^{k_{j}}{d_{i}^{(j+1)}F^{(j)}(y_{0})}+O(h^{p+1}), \\ e'_{n+1}=y'(t_{0}+h)-y'_{1}=\sum_{j=1}^{p}h^{j}\sum_{i=1}^{k_{j}}{{d'}_{i}^{(j)}F^{(j)}(y_{0})}+O(h^{p+1}), \end{cases} $$

where $F^{(j)}(y_{0})$ denotes an elementary differential and the terms $d_{i}^{(j+1)}$ and ${d'}_{i}^{(j)}$ depend on the coefficients of the EFRKN method. Method (2) has order *p* if, for every sufficiently smooth IVP (1) and for any small step size *h*, the local truncation errors of the numerical solutions satisfy

$$ \textstyle\begin{cases} e_{1}=y(t_{0}+h)-y_{1}=O(h^{p+1}), \\ e'_{1}=y'(t_{0}+h)-y'_{1}=O(h^{p+1}), \end{cases} $$

or equivalently,

$$ \textstyle\begin{cases} d_{i}^{(j+1)}=0,\quad i=1,\ldots,k_{j},j=1,\ldots,p-1, \\ {d'}_{i}^{(j)}=0,\quad i=1,\ldots,k_{j},j=1,\ldots,p. \end{cases} $$

For the EFRKN method, the coefficients are dependent on *z*. Specifically, they are even functions of *z*. Thus, we use the following Taylor expansions:

$$\begin{aligned} &\bar{b}_{i}(h)=\bar{b}_{i}^{(0)}+ \bar{b}_{i}^{(2)}h^{2}+\bar {b}_{i}^{(4)}h^{4}+ \cdots, \quad\quad b_{i}(z)=b_{i}^{(0)}+b_{i}^{(2)}h^{2}+b_{i}^{(4)}h^{4}+ \cdots, \\ &\gamma_{i}(z)=1+\gamma_{i}^{(2)}h^{2}+ \gamma _{i}^{(4)}h^{4}+\cdots, \quad\quad a_{ij}(z)=a_{ij}^{(0)}+a_{ij}^{(2)}h^{2}+a_{ij}^{(4)}h^{4}+ \cdots. \end{aligned}$$

Using these assumptions and following the way given in [14] (pp. 143–148), we obtain the terms of the local truncation error. Roughly speaking, comparing the Taylor expansions of $y_{1}$, $y_{1}'$ in (2) with and the Taylor expansions of $y(t_{0}+h)$, $y'(t_{0}+h)$ at $t_{0}$, the order *k* condition can be obtained by making the coefficients of $h^{i}$, $i=1,\ldots,k$, equal. Following this procedure, the order conditions (up to the fifth order) for the EFMRKN methods considered in this paper can be obtained.

Order 1 requires:

$$ {d'}_{1}^{(1)}:=\sum _{i}b_{i}^{(0)}-1=0. $$

Order 2 requires in addition:

$$ \begin{aligned} {d'}_{1}^{(2)}:=\sum _{i}b_{i}^{(0)}c_{i}- \frac{1}{2}=0,\quad \quad d_{1}^{(2)}:=\sum _{i}\bar{b}_{i}^{(0)}- \frac{1}{2}=0. \end{aligned} $$

Order 3 requires in addition:

$$ \begin{aligned} &{d'}_{1}^{(3)}:= \sum_{i}b_{i}^{(0)}c_{i}^{2}- \frac {1}{3}=0,\quad\quad {d'}_{2}^{(3)}:=\sum _{i}b_{i}^{(0)}\sum _{k}a_{ik}^{(0)}-\frac{1}{6}=0, \\ &{d'}_{3}^{(3)}:=\sum _{i}b_{i}^{(2)}=0,\quad\quad d_{1}^{(3)}:=\sum_{i} \bar{b}_{i}^{(0)}c_{i}-\frac{1}{6}=0. \end{aligned} $$

Order 4 requires in addition:

$$ \begin{aligned} &{d'}_{1}^{(4)}:= \sum_{i}b_{i}^{(0)}c_{i}^{3}- \frac{1}{4}=0, \quad\quad {d'}_{2}^{(4)}:=\sum _{i}b_{i}^{(0)}\sum _{k}c_{i}a_{ik}^{(0)}-\frac{1}{8}=0, \\ &{d'}_{3}^{(3)}:=\sum _{i}b_{i}^{(0)}\sum _{k}a_{ik}^{(0)}c_{k}-\frac{1}{24}=0, \quad\quad {d'}_{4}^{(4)}:=\sum _{i}b_{i}^{(0)}c_{i}\gamma _{i}^{(2)}=0, \\ &{d'}_{5}^{(4)}:=\sum _{i}b_{i}^{(2)}c_{i}=0, \quad \quad d_{1}^{(4)}:=\sum_{i}\bar {b}_{i}^{(0)}c_{i}^{2}- \frac{1}{12}=0, \\ &{d}_{2}^{(4)}:=\sum_{i} \bar{b}_{i}^{(0)}\sum_{k}a_{ik}^{(0)}- \frac{1}{24}=0, \quad\quad {d}_{3}^{(4)}:=\sum _{i}\bar{b}_{i}^{(2)}=0. \end{aligned} $$

Order 5 requires in addition:

$$\begin{aligned} &{d'}_{1}^{(5)}:= \sum_{i}b_{i}^{(0)}c_{i}^{4}- \frac{1}{5}=0, \quad\quad {d'}_{2}^{(5)}:=\sum _{i}b_{i}^{(0)}\sum _{k}c_{i}^{2}a_{ik}^{(0)}- \frac{1}{10}=0, \\ &{d'}_{3}^{(5)}:=\sum _{i}b_{i}^{(0)}c_{i}\sum _{k}a_{ik}^{(0)}c_{k}- \frac{1}{30}=0, \quad\quad {d'}_{4}^{(5)}:=\sum _{i}b_{i}^{(0)}\sum _{k}a_{ik}^{(0)}c_{k}^{2}- \frac{1}{60}=0, \\ &{d'}_{5}^{(5)}:=\sum _{i}b_{i}^{(2)}c_{i}^{2}=0, \quad\quad {d'}_{6}^{(5)}:=\sum _{i}b_{i}^{(0)}\sum _{k}a_{ik}^{(2)}c_{k}^{2}=0, \\ &{d'}_{7}^{(5)}:=\sum _{i}b_{i}^{(4)}=0, \quad\quad d_{1}^{(5)}:=\sum_{i}\bar {b}_{i}^{(0)}c_{i}^{3}- \frac{1}{20}=0, \\ &{d}_{2}^{(5)}:=\sum_{i} \bar{b}_{i}^{(0)}c_{i}\sum _{k}a_{ik}^{(0)}-\frac{1}{10}=0, \quad \quad {d}_{3}^{(5)}:=\sum_{i} \bar{b}_{i}^{(2)}c_{i}=0, \\ &{d}_{4}^{(5)}:=\sum_{i} \bar{b}_{i}^{(0)}\sum_{j} \bar{a}_{ij}^{(0)}c_{j}=0. \end{aligned}$$

## Construction of implicit symmetric and symplectic modified EFRKN methods

Based on the above conditions, in this section, we will construct an implicit EFMRKN method under the symmetry, symplecticity, exponential fitting conditions obtained in the previous two sections. We consider a brief case $s=2$ in which there will not be so many coefficients.

For $s=2$, the symmetry conditions (3) and the symplecticity conditions (9) are specified as

16 $$ \begin{gathered} c_{1}+c_{2}=1, \quad\quad b_{1}=b_{2},\quad\quad \bar{b}_{1}+\bar {b}_{2}=b_{1},\quad\quad c_{1} \gamma_{1}+c_{2}\gamma_{2}=1, \\ a_{12}=a_{21}+b_{1}(c_{1} \gamma_{1}-c_{2}\gamma_{2}), \quad \quad a_{21}=a_{12}+b_{2}(c_{2} \gamma_{2}-c_{1}\gamma_{1}), \\ \bar{b}_{1}=b_{1}c_{2}\gamma_{2},\quad \quad \bar {b}_{2}=b_{2}c_{1}\gamma_{1}, \quad\quad b_{1}\bar {b}_{2}-b_{1}a_{12}=b_{2} \bar{b}_{1}-b_{2}a_{21}. \end{gathered} $$

As we can see, there is an equation $c_{1}+c_{2}=1$. By introducing another parameter *θ*, it will come to $c_{1}=\frac{1}{2}-\theta $, $c_{2}=\frac{1}{2}+\theta$. There is no constraint on *θ* and it can take any real value. After this, equations (16) can be in a simplified form

17 $$ \begin{gathered} c_{1}=\frac{1}{2}-\theta, \quad\quad c_{2}=\frac{1}{2}+\theta, \quad\quad b_{2}=b_{1}, \quad\quad \bar{b}_{1}=b_{1}c_{2} \gamma_{2}, \quad\quad \bar{b}_{2}=b_{1}c_{1} \gamma_{1}, \\ a_{21}-a_{12}=b_{1}(c_{2} \gamma_{2}-c_{1}\gamma_{1}), \quad \quad 1=c_{1}\gamma_{1}+c_{2}\gamma_{2}. \end{gathered} $$

Combining $\bar{b}_{1}=b_{1}c_{2}\gamma_{2}$, $\bar {b}_{2}=b_{1}c_{1}\gamma_{1}$, $a_{21}-a_{12}=b_{1}(c_{2}\gamma _{2}-c_{1}\gamma_{1})$, and EF conditions (11) (when $s=2$), we obtain

18 $$\begin{aligned} &b_{1}=\frac{\sinh(z/2)}{z\cosh(\theta z)}, \end{aligned}$$

19 $$\begin{aligned} &b_{1}(c_{1}\gamma_{1}-c_{2} \gamma_{2}) =\frac{2\sinh(z/2)-z\cosh (z/2)}{z^{2}\sinh( \theta z)}, \end{aligned}$$

20 $$\begin{aligned} &(a_{11}+a_{12})\cosh(\theta z)=\frac{\cosh(\theta z)-\cosh (z/2)+c_{1}\gamma_{1}z\sinh(z/2)}{z^{2}}, \end{aligned}$$

21 $$\begin{aligned} &(a_{12}-a_{11})\sinh(\theta z)=\frac{\sinh(z/2)-\sinh( \theta z)-c_{1}\gamma_{1}z\cosh(z/2)}{z^{2}}, \end{aligned}$$

22 $$\begin{aligned} &(a_{21}+a_{22})\cosh(\theta z)=\frac{\cosh(\theta z)-\cosh (z/2)+c_{2}\gamma_{2}z\sinh(z/2)}{z^{2}}, \end{aligned}$$

23 $$\begin{aligned} &(a_{22}-a_{21})\sinh(\theta z)=\frac{\sinh(z/2)+\sinh( \theta z)-c_{2}\gamma_{2}z\cosh(z/2)}{z^{2}}. \end{aligned}$$

Using $c_{1}\gamma_{1}+c_{2}\gamma_{2}=1$ in equations (18) and (19), it is easy to obtain

24 $$ \gamma_{1}=\frac{1}{2c_{1}}+\frac{2 \sinh(z/2)-z\cosh (z/2)}{2c_{1}b_{1}z^{2}\sinh( \theta z)}. $$

The Taylor expansion of $\gamma_{1}$ is

$$ \gamma_{1}=\frac{1-6\theta}{12\theta^{2}-6\theta }+ \frac{-1+20\theta^{2}}{-360\theta+720\theta^{2}}z^{2}+ \cdots. $$

As is pointed out in Sect. 3, when $z\rightarrow0$, $\gamma _{1}(z)\rightarrow1$, we have

$$ 1=\frac{1-6\theta}{12\theta^{2}-6\theta}, \quad {\text{i.e.}}, \quad \theta=\pm\frac{\sqrt{3}}{6}. $$

Thanks to the classical SSRKN method in [24], $a_{11}=a_{22}$ is picked in this paper. Under this assumption, by simple calculation, it is not strenuous to find the following equalities: (22) − (20) ⇒ (18), (21) + (23) ⇒ (19). So, method (2) satisfying (18), (19), (20), and (21) is exponentially fitted. In (20) and (21), only $a_{11}$ and $a_{12}$ are unknown. By adding and subtracting, the expressions of $a_{11}$ and $a_{12}$ are

$$ a_{11}=\frac{\sinh(2\theta z)-\sinh (c_{2}z)+c_{1} \gamma_{1}z\cosh(c_{2}z)}{z^{2}\sinh(2\theta z)}, \quad \quad a_{12}=\frac{\sinh(c_{1}z)-c_{1} \gamma_{1}z\cosh (c_{1}z)}{z^{2}\sinh(2\theta z)}. $$

Until now, we have obtained an implicit symmetric and symplectic exponentially fitted Runge–Kutta–Nyström method whose coefficients are given by

25 $$\begin{aligned} \begin{gathered} \theta=\pm\frac{\sqrt{3}}{6}, \quad \quad c_{1}=\frac{1}{2}-\theta ,\quad\quad c_{2}=\frac{1}{2}+\theta,\quad\quad \gamma_{1}=\frac {1}{2c_{1}}+ \frac{2\sinh(z/2)-z\cosh(z/2)}{2c_{1}b_{1}z^{2} \sinh (\theta z)}, \\ b_{1}=\frac{\sinh(z/2)}{z\cosh(\theta z)}, \quad\quad b_{2}=b_{1}, \quad\quad \bar{b}_{1}=b_{1}(1-c_{1} \gamma_{1}), \quad \quad \bar{b}_{2}=b_{2}c_{1} \gamma_{1}, \\ a_{11}=\frac{\sinh(2\theta z)-\sinh(c_{2}z)+c_{1} \gamma _{1}z\cosh(c_{2}z)}{z^{2}\sinh(2\theta z)}, \quad\quad a_{22}=a_{11}, \\ a_{12}=\frac{\sinh(c_{1}z)-c_{1} \gamma_{1}z\cosh (c_{1}z)}{z^{2}\sinh(2\theta z)}, \quad\quad a_{21}=a_{12}+b_{1}(1-2c_{1} \gamma_{1}). \end{gathered} \end{aligned}$$

We denote this method as ISSEFMRKN2. For small values of *z*, the series expansions for the coefficients are given by

$$\begin{aligned}& b_{1}=\frac{1}{2}+\biggl(\frac{1}{48}-\frac{\theta^{2}}{4} \biggr)z^{2}+\biggl(\frac {1}{3840}-\frac{\theta^{2}}{96}+ \frac{5\theta^{4}}{48}\biggr)z^{4}+\cdots, \\& \gamma_{1}=\frac{1-6\theta}{12\theta^{2}-6\theta}+\frac {-1+20\theta^{2}}{-360\theta+720\theta^{2}}z^{2}+ \cdots, \\& \gamma_{2}=\frac{1+6\theta}{12\theta^{2}+6\theta}+\frac {-1+20\theta^{2}}{360\theta+720\theta^{2}}z^{2}+ \cdots, \\& \bar{b}_{1}=\frac{1+6\theta}{24\theta}+\frac{3+30\theta -20\theta^{2}-360\theta^{3}}{2880\theta}z^{2}+ \cdots, \\& \bar{b}_{2}=\frac{6\theta-1}{24\theta}+\frac{-3+30\theta +20\theta^{2}-360\theta^{3}}{2880\theta}z^{2}+ \cdots, \\& a_{11}=\frac{-13+160\theta^{2}+720\theta^{4}}{2880\theta ^{2}}+\frac{-37+3052\theta^{2}-27{,}888\theta^{4}-100{,}800\theta ^{6}}{967{,}680\theta^{2}}z^{2}+\cdots, \\& \begin{aligned} a_{12}&=\frac{13-120\theta+200\theta^{2}+720\theta ^{4}}{2880\theta^{2}} \\ &\quad{} +\frac{40{,}357-1008\theta-479{,}332\theta ^{2}+6720\theta^{3}-32{,}592\theta^{4}-100{,}800\theta^{6}}{967{,}680\theta ^{2}}z^{2} +\cdots, \end{aligned} \\& \begin{aligned} a_{21}&=\frac{13+120\theta+200\theta^{2}+720\theta ^{4}}{2880\theta^{2}} \\ &\quad{}+\frac{40{,}357+1008\theta-479{,}332\theta ^{2}-6720\theta^{3}-32{,}592\theta^{4}-100{,}800\theta^{6}}{967{,}680\theta ^{2}}z^{2}+\cdots. \end{aligned} \end{aligned}$$

From the Taylor expansions, we can verify that our method ISSEFMRKN2 meets all the four order conditions, but fails in the fifth order condition ${d'}_{1}^{(5)}:=\sum_{i}b_{i}^{(0)}c_{i}^{4}-\frac{1}{5}=0$. So, the method ISSEFMRKN2 is of order 4. When $z\rightarrow0$, ISSMEFRKN2 reduces to SSRKN of order 4 in [24] with $a_{11}=\frac{1}{45}$.

## Numerical experiments

To test the numerical performance of the method ISSEFMRKN2, we carry out experiments on four problems to illustrate the effectiveness and efficiency. The codes used for comparison are

- DIRKNRaed: The embedded diagonally implicit RKN 4(3) pair method proposed by Al-Khasawneh et al. in [1].
- DIRKNNora: The three-stage fourth-order diagonally implicit RKN method proposed by Senu et al. in [27].
- ISSRKN2: The symmetric and symplectic two-stage fourth-order implicit RKN method proposed by MENG-ZHAO QIN et al. in [24] with $a_{11}=\frac{-13+160\theta^{2}+720\theta^{4}}{2880\theta^{2}}$ and $\theta=\pm\frac{\sqrt{3}}{6}$, i.e., $a_{11}=\frac{1}{45}$.
- ISSEFRKN2: The symmetric and symplectic exponentially fitted two-stage RKN method proposed in [35] which is of order 4.
- ISSEFMRKN2: The symmetric and symplectic exponentially fitted two-stage modified RKN method (25) proposed in this paper which is of order 4.

As we can see, the proposed method ISSEFMRKN2 is implicit. The main computation is in computing the non-linear equations

$$ \textstyle\begin{cases} Y_{1}=y_{0}+c_{1}\gamma _{1}h{y'_{0}}+h^{2}(a_{11}f(t_{0}+c_{1}h,Y_{1})+a_{12}f(t_{0}+c_{2}h,Y_{2})), \\ Y_{2}=y_{0}+c_{2}\gamma _{2}h{y'_{0}}+h^{2}(a_{21}f(t_{0}+c_{1}h,Y_{1})+a_{22}f(t_{0}+c_{2}h,Y_{2})). \end{cases} $$

In this paper, we use the Newton iteration method with initial values $Y^{(0)}_{1}=Y^{(0)}_{2}=y(0)$. Here we use two stopping criteria for iteration. First, iterations are carried out until the difference between the Euclidean norm of two successive iterations attains 10^−8^. Second, if the first criterion fails, then the iteration will be forced to terminate after running 1000 times.

**Figure 1:**
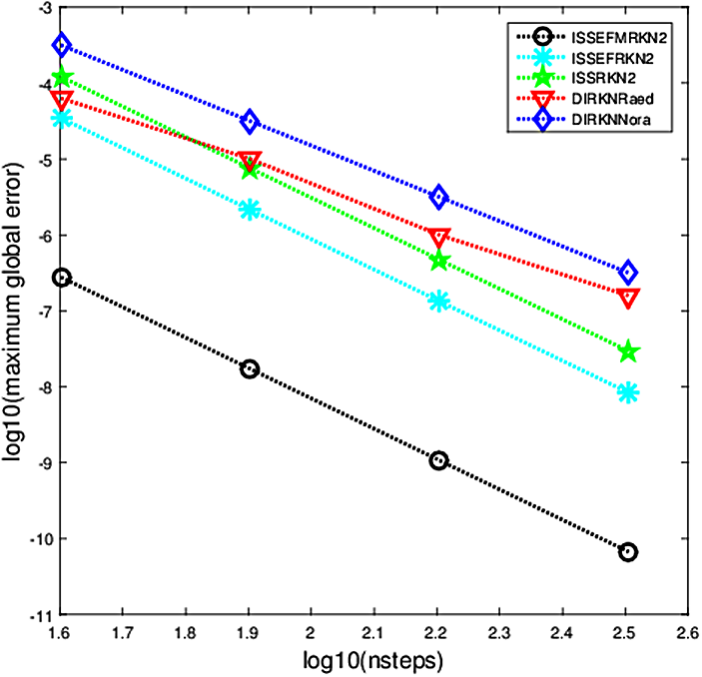
Maximum global error in the solution for Problem 1

**Figure 2:**
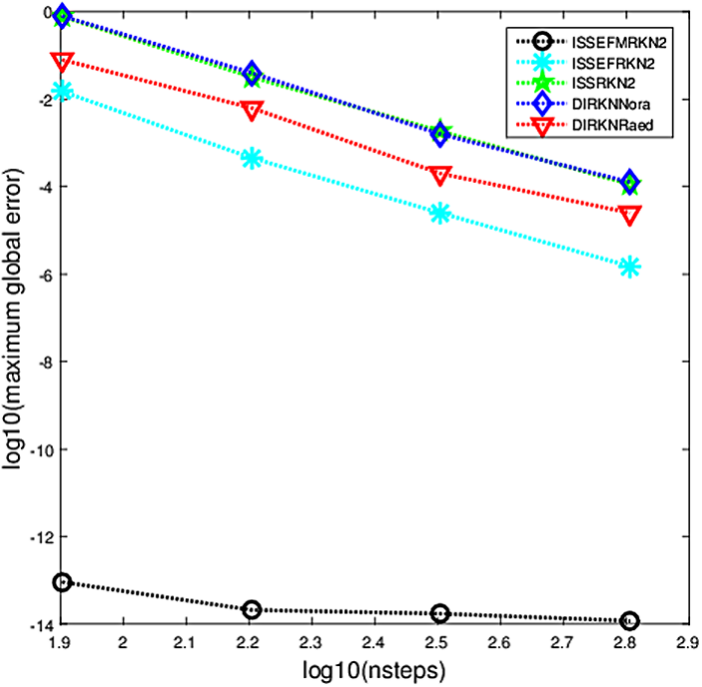
Maximum global error in the solution for Problem 2

**Figure 3:**
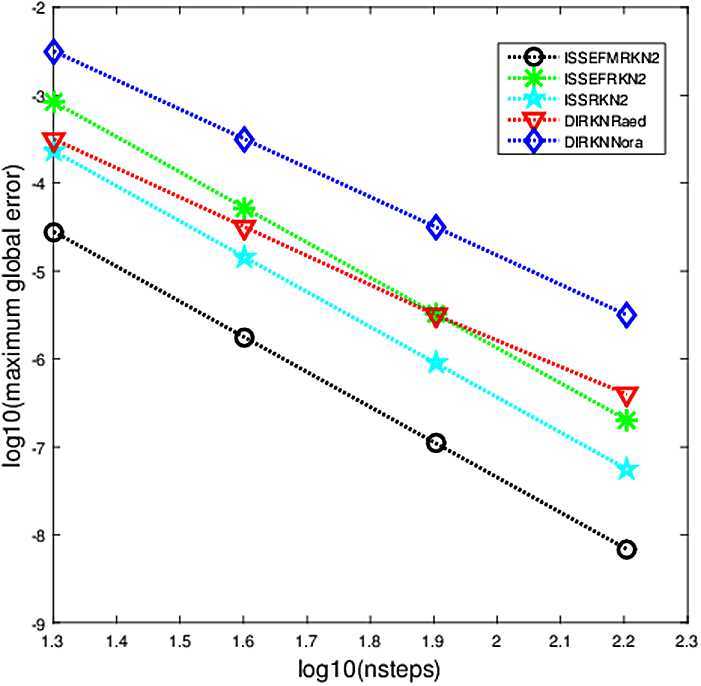
Maximum global error in the solution for Problem 3 with $\lambda=1.2i$

**Figure 4:**
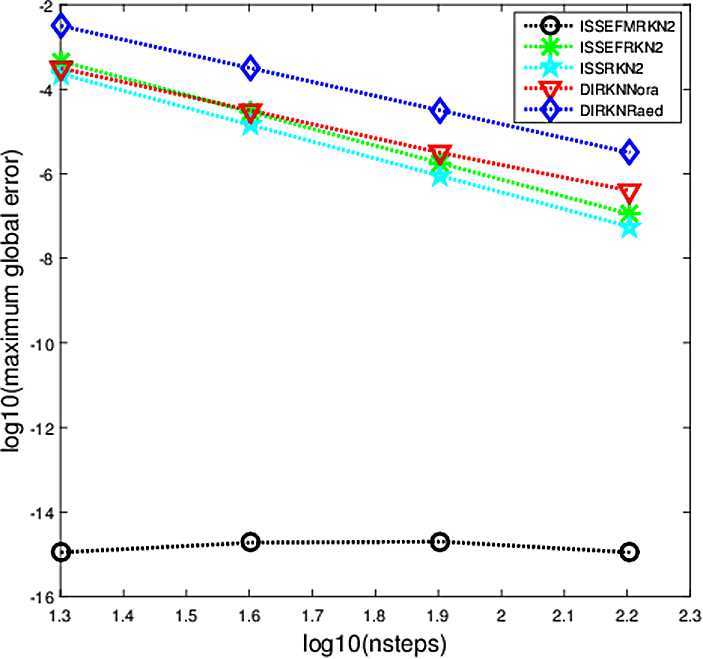
Maximum global error in the solution for Problem 3 with $\lambda=i$

**Figure 5:**
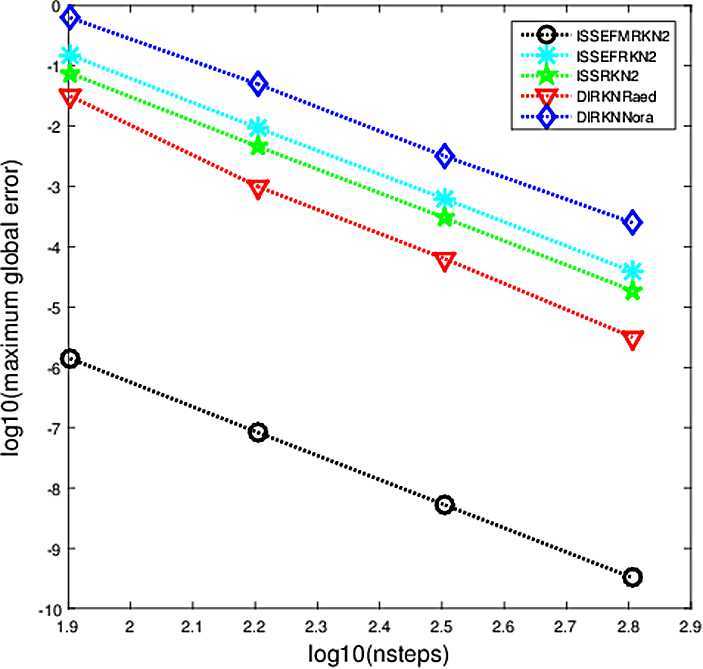
Maximum global error in the solution for Problem 4

The criterion used in the numerical comparisons is the usual test based on computing the maximum global error in the solution over the whole integration interval. In Figs. 1–5, we show the decimal logarithm of the maximum global error (log10(err)) versus the number of steps required by each code on a logarithmic scale (log10(nsteps)). All computations are carried out in MATLAB(Version R2015b), on a notebook computer with Intel Core(TM)i7-2640M CPU (2.80 GHz) and 8 GB RAM.

### Problem 1

We consider the perturbed orbital problem (studied in [11])

$$ \textstyle\begin{cases} y_{1}''=-\frac{y_{1}}{(y_{1}^{2}+y_{2}^{2})^{3/2}}-\frac {(2\varepsilon+\varepsilon ^{2})y_{1}}{(y_{1}^{2}+y_{2}^{2})^{5/2}},\quad t\in[0,t_{\mathrm {end}}], \\ y_{2}''=-\frac{y_{2}}{(y_{1}^{2}+y_{2}^{2})^{3/2}}-\frac {(2\varepsilon+\varepsilon^{2})y_{2}}{(y_{1}^{2}+y_{2}^{2})^{5/2}}, \\ y_{1}(0)=1,\quad\quad y_{1}'(0)=0, \quad\quad y_{2}(0)=0, \quad \quad y_{2}'(0)=1+\varepsilon, \end{cases} $$

whose exact solution is

$$ y_{1}(t)=\cos(t+\varepsilon t), \quad\quad y_{2}(t)=\sin (t+\varepsilon t). $$

In our test we choose $\omega=1$, $\lambda=i$, $t_{\mathrm{end}}=10$, and the parameter values $\varepsilon= 10^{-3}$, and the numerical results presented in Fig. 1 have been computed with the integration steps $h=1/2^{m}$, $m=2,3,4,5$.

### Problem 2

Consider the second-order ODE

$$ \textstyle\begin{cases} y''=-30\sin(30t), \quad t \in[0, t_{\mathrm{end}}], \\ y(0)=0, \quad\quad y'(0)=1, \end{cases} $$

whose analytic solution is given by

$$ y(t)=\sin(30t)/30. $$

For this problem, the parameters are chosen as $\omega=30$, $\lambda =30i$, $t_{\mathrm{end}}=10$, $h=1/2^{m}$, $m=3,4,5,6$. The numerical result can be seen in Fig. 2.

### Problem 3

We consider the second-order ODEs

$$ \textstyle\begin{cases} y_{1}''=(\mu-2)y_{1}+(2\mu-2)y_{2}, \quad t\in[0,t_{\mathrm {end}}] \\ y_{2}''=(1-\mu)y_{1}+(1-2\mu)y_{2}, \\ y_{1}(0)=2, \quad\quad y_{1}'(0)=0, \quad\quad y_{2}(0)=-1, \quad \quad y_{2}'(0)=0, \end{cases} $$

where *μ* is an arbitrary parameter. The exact solution is

$$ y_{1}(t) =2\cos(t), \quad\quad y_{2}(t)=-\cos(t). $$

In this problem we chose the parameters as follows: $\mu=1.44$, $\lambda=1.2i$ (Fig. 3), $\lambda=i$ (Fig. 4), $t_{\mathrm{end}}=10$. For the integration steps *h*, we select them as $h=1/2^{m}$, $m=1,2,3,4$. The numerical result is presented in Figs. 3–4, one each for $\lambda=1.2i$, $\lambda=i$.

### Problem 4

We consider the coupled linear system (studied in [11])

$$ \textstyle\begin{cases} y_{1}''=-\frac{101}{2}y_{1}+\frac{99}{2}y_{2}+\varepsilon(\frac {93}{2}\cos(2t)-\frac{99}{2}\sin(2t)), \quad t\in[0, t_{\mathrm {end}}], \\ y_{2}''=\frac{99}{2}y_{1}-\frac{101}{2}y_{2}+\varepsilon(\frac {93}{2}\sin(2t)-\frac{99}{2}\cos(2t)), \\ y_{1}(0)=-1+\varepsilon, \quad\quad y_{1}'(0)=-10, \quad\quad y_{2}(0)=1, \quad\quad y_{2}'(0)=10+2\varepsilon, \end{cases} $$

whose exact solution is

$$\begin{aligned} &y_{1}(t) =-\cos(10t)-\sin(10t)+\varepsilon\cos(2t), \\ &y_{2}(t)=\cos(10t)+\sin(10t)+\varepsilon\sin(2t). \end{aligned}$$

This solution represents a periodic motion with different frequencies. In our test we choose the parameter values $\lambda=10i$, $t_{\mathrm{end}}=10$, $\varepsilon= 10^{-3}$, and the numerical results stated in Fig. 5 have been computed with steps $h=1/2^{m}$, $m=3,4,5,6$.

From Figs. 1–5, we can find that the implicit modified EFRKN method ISSEFMRKN2 is more efficient than ISSEFRKN2 and the symmetric and symplectic method ISSRK2. ISSEFRKN2 does not possess higher accuracy than ISSRKN2 for Problems 3 and 4. For Problem 3, its true frequency is 1. In the numerical study, we select two frequencies 1.2 (Fig. 3) and 1 (Fig. 4). From Figs. 3–4, we can see the accuracies are quite different. The accuracy of 1 is much higher than that of 1.2. In this problem, we know its true frequency. But when it comes to applications, the true frequency is often unpredictable. Therefore, we need to try some different candidates. For Problems 2 and 3, we find that ISSEFMRKN2 is much more accurate and efficient than our methods considered in this paper. As is pointed out in introduction, ISSEFRKN2 is not a complete EF method. However, ISSEFMRKN2 is completely EF. The solutions of Problems 2 and 3 are all in the form of a triangular function. This is just in line with EF methods. So, ISSEFMRKN2 performs very well.

## Conclusions

In this paper a two-stage symmetric, symplectic IEFMRKN integrator has been derived. Like the existing EFRKN integrators such as [34, 35], the coefficients of the new method depend on the product of the dominant frequency *ω* and the step size *h*. When the parameter *z* approaches zero, the ISSEFMRKN2 method reduces to the classical RKN method. The numerical experiments carried out show that the new method is more efficient than some implicit RKN methods. In every experiment, the method ISSEFMRKN2 is shown to be the most efficient one among the methods used for comparison. However, like the ISSEFRKN2 method in [35], we derive only one method, not a class of methods whose coefficients can be dependent on one or more parameters. In the future, we will consider deriving a class of ISSEFRKN methods.
